# Curcumin and its formulations for the treatment of polycystic ovary syndrome: current insights and future prospects

**DOI:** 10.1186/s13048-025-01660-z

**Published:** 2025-04-15

**Authors:** Pooja Mallya, Shaila A. Lewis

**Affiliations:** Department of Pharmaceutics, Manipal College of Pharmaceutical Sciences, Manipal Academy of Higher Education (MAHE), Manipal, Karnataka 576104 India

**Keywords:** Polycystic ovary syndrome, Curcumin, Formulations, Combination, Phytochemicals

## Abstract

Polycystic ovary syndrome (PCOS) is a common gynaecological complication with alarmingly high incidence of 6–20% in women of reproductive age and leads to multifaceted symptoms such as menstrual irregularities, hyperandrogenism, polycystic ovaries, and insulin resistance. Several therapeutic methods have been recommended for PCOS including lifestyle modification, insulin sensitizer (metformin), ovulation inducers (letrozole, clomiphene citrate), hormonal pills, and surgical intervention (ovarian drilling and oophorectomy); however, these treatment modalities often cause adverse effects. Currently, phytochemicals and plant extracts have been recommended for PCOS. Among these, few phytochemicals and their formulations, curcumin (CUR) (a bioactive polyphenol from *Curcuma longa*), has emerged as a promising complementary PCOS therapy due to its antioxidant, anti-inflammatory, insulin-sensitizing, and ovulation inducing properties. However, CUR's clinical application is hindered by poor solubility and bioavailability. In this review, we summarize and discuss various formulations of CUR and combination therapies that have demonstrated potential in treating PCOS in animal models.

## Introduction

**Fig. 1:**
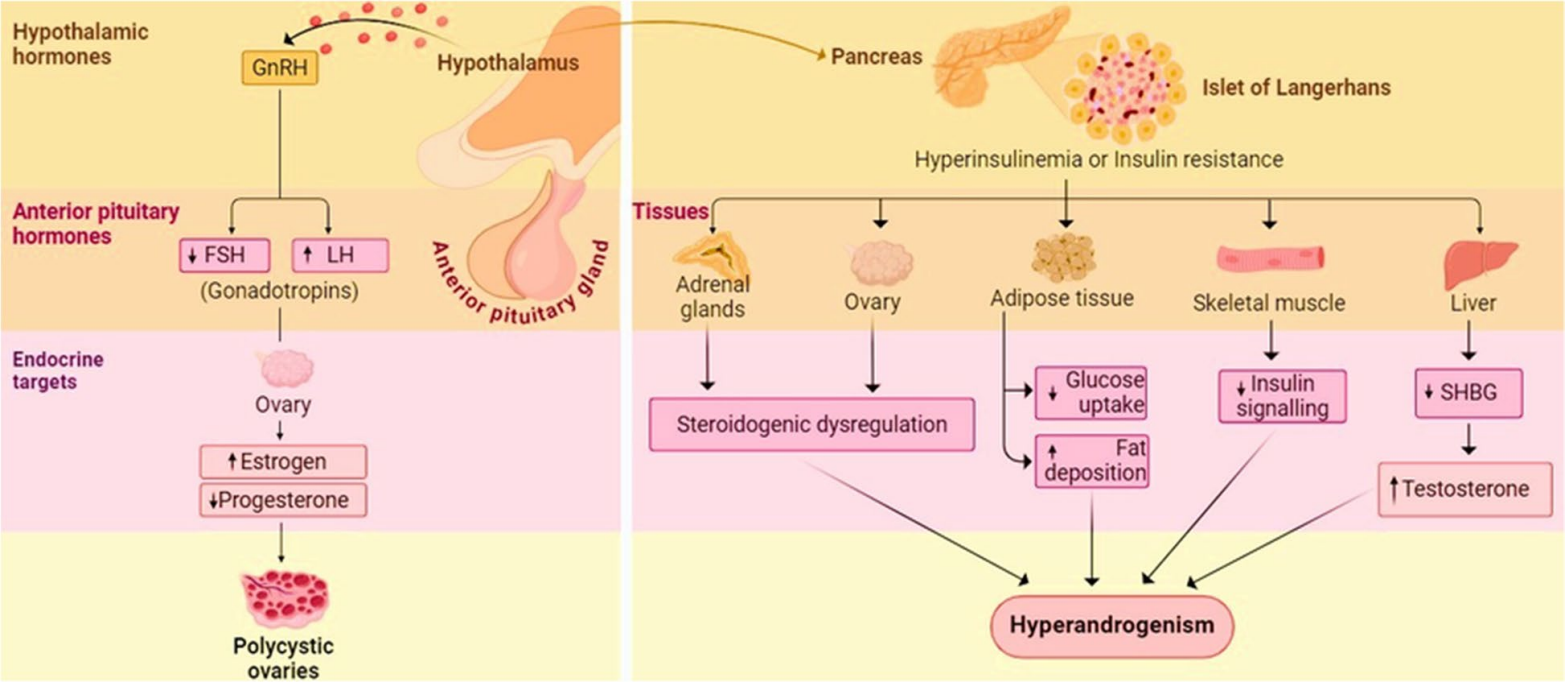
Hypothalamic pituitary ovarian (HPO) axis dysfunction increasing pulsatile secretion of gonadotropin releasing hormone and insulin leading to the formation of polycystic ovaries and hyperandrogenism

Polycystic ovary syndrome (PCOS) is a heterogeneous condition affecting 6—20% of women of reproductive age, characterized by anovulation, metabolic and endocrine disorders [1]. Its complex pathophysiology is due to cumulative facets such as hypothalamic pituitary ovarian (HPO) axis dysfunction, steroidogenic pathway and neuroendocrine dysfunctions, oxidative stress, inflammation, insulin resistance, hyperinsulinemia, genetics, and environmental factors [2]. The diagnosis and treatment often revolve around hyperinsulinemia and hyperandrogenism **(**Fig. 1**)** [3, 4]. Women with PCOS exhibit a wide variety of clinical and biochemical manifestations including menstrual irregularities, polycystic ovaries [5], hyperandrogenism, type II diabetes, low-grade inflammation, obesity, dyslipidaemia, insulin resistance (IR) [6], impaired glucose tolerance [7], and infertility [8].

### Current treatment approaches

PCOS treatment arrays from lifestyle modifications to pharmacological interventions, with lifestyle changes recommended for mild symptoms. Pharmacological treatment is tailored to individual needs based on severity of symptoms. Commonly used treatments for PCOS are summarized in Table 1 [5]. Clomiphene citrate, an ovulation inducer causes enlarged ovaries, ovarian hyperstimulation syndrome (OHSS), and multiple pregnancies. About 30% of the overweight women may not respond to this therapy, and it can reduce conception rates by 20% due to its antiestrogenic effect on cervical mucus and the endometrium [9]. Long-term use of oral contraceptives can lead to weight gain, venous thrombosis, cancer, and impacts on bone health. Surgical procedures, such as laparoscopy or laparotomy may be necessary if the cysts persist, though they possess the risk of ovarian torsion. Although these agents address PCOS symptoms, they have neither been approved by the FDA nor EMA for PCOS treatment [10, 11].

**Table 1:** PCOS treatment strategies

Treatment	Therapeutic intervention	Indication	Adverse effects
First-line therapy	Lifestyle modification (Weight loss, exercise, and diet)	Improved body weight, fertility, hormonal profile, and insulin levels	Not effective against moderate to severe symptoms of PCOS [12]
Second-line therapy	MTF (Insulin sensitizer)	Inhibits glucose production in liver, increases peripheral insulin sensitivity and glucose uptake	Nausea, headache, low Vitamin B12, GI disturbance, arrhythmia, cold skin, congenital malformation, and ovarian hyperstimulation syndrome (OHSS) [9, 13]
Third-line therapy	Dipeptidyl peptidase- 4 inhibitors- vildagliptin, sitagliptin, alogliptin, linagliptin, and saxagliptin	Improves insulin sensitivity, hyperandrogenism, and hyperglycemia	Breathing difficulty, headache, hypersensitivity reactions, and pharyngitis [14]

### Objectives

- First line drugs such as Metformin (MTF) and Clomiphene citrate are associated with adverse effects such as multiple pregnancies, Ovary hyperstimulation syndrome, and gastric disturbances
- Phytochemicals are widely used alternatives among which CUR is widely reported and known to have similar effect to MTF
- Formulations of CUR in the PCOS treatment have a potential in alleviating the symptoms of PCOS

### Need for complementary therapies

Preclinical investigations have established the potential of herbal drugs in treating PCOS, with polyphenols such as CUR, rutin, quercetin, catechin, gallic acid, cinnamon, and resveratrol showing effectiveness [15]. Emerging research indicates CUR, a polyphenol derived from *Curcuma longa* rhizomes to be potential in treating PCOS [16]. However, its therapeutic application is often limited by poor permeability as it is a P-glycoprotein (P-gp) efflux substrate, low aqueous solubility (< 1 g/ml), as well as poor oral bioavailability (< 1%) due to extensive phase II metabolism in the presence of uridine 5′-diphosphate-glucuronosyltransferase (UGT) and β-glucuronidase enzymes [17]. This review discusses various formulation approaches and combination therapies of CUR for PCOS treatment with a special focus on patents and future prospects. This review was compiled focusing on literature from past decade. Online databases-Scopus, Science Direct, Google scholar, and PubMed using multiple keywords combinations were explored for literature review. The keywords included “PCOS”, “curcumin”, “formulations”, “combination”, and “phytochemicals”. The literature search was conducted and suitable articles were selected based on relevance to the topic.

## CUR and PCOS: Focusing on mechanism

**Fig. 2:**
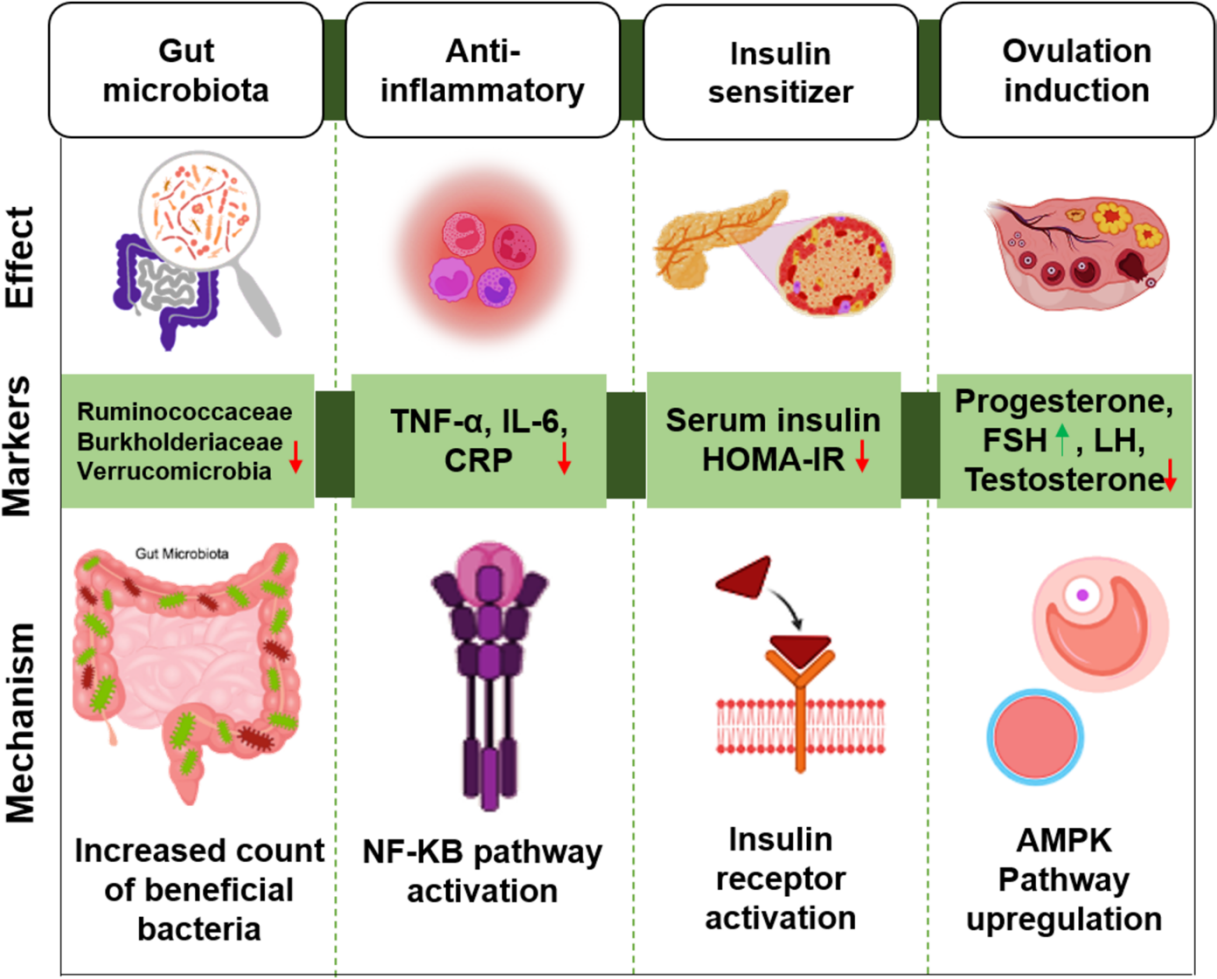
CUR effects and mechanism for the treatment of PCOS

CUR targets multiple receptors and signalling pathways involved in PCOS (Fig. 2). The antioxidant and anti-inflammatory effect of CUR in ovarian tissues, phytoestrogenic effect, ovulation induction, and the modulation effect of CUR on insulin, androgens, glucose, and lipids are beneficial for treating PCOS with similar effectiveness as MTF [18–20].

### CUR and NF-κB pathway

CUR affects multiple molecular pathways, including the NF-κB pathway, which plays a central role in inflammation, cancer, and other diseases. CUR inhibits NF-κB, a key regulator of genes involved in cell growth, inflammation, and adhesion. It suppresses NF-κB activity in a dose-dependent manner (1–100 µM), reducing cytokine and chemokine levels in cell cultures. Animal studies show that CUR, both orally and parenterally, effectively blocks NF-κB-dependent cellular processes. CUR inhibits NF-κB activation by blocking IKK activity, preventing IκB phosphorylation, and inhibiting NF-κB translocation to the nucleus. This inhibition is a major mechanism through which CUR reduces COX- 2 gene expression and its subsequent protein transcription [21]. The activation of NF-κB leads to the release of pro-inflammatory cytokines, causing low-grade systemic and ovarian inflammation, disrupting follicular development, and contributing to the onset of PCOS. In a study with C57BL/6 J mice, the animals were divided into four groups: control, control with CUR, DHEA induced PCOS model, and CUR treated DHEA group. After 45 days of CUR intervention (200 mg/kg), PCOS symptoms were alleviated by inhibiting the NF-κB pathway and reducing intestinal permeability, as indicated by statistically significant results (P < 0.05) [22]. CUR inhibits NF-кB, Jun N-terminal kinase (JNK) and extracellular-signal regulated kinase (ERK 1 or 2) signalling pathways thereby reducing necrotic hepatocyte count, interleukin- 6, TNF-α, and C-reactive protein levels [23].

### CUR and AMPK activation

CUR activates both the MAPK (mitogen-activated protein kinase) and AMPK (AMP- activated protein kinase) signalling pathways, enhancing glucose uptake [24]. Through AMPK activation, CUR suppresses gluconeogenesis in hepatocytes by inhibiting glucose- 6-phosphatase and phosphoenolpyruvate-carboxykinase, while also promoting Glucose transported-type 4 (GLUT4) translocation and glucose uptake in adipocytes [25]. CUR also stimulates p38-MAPK, which is crucial for glucose uptake in response to insulin. Research has shown that CUR (10 µM) activates p38-MAPK in a time-dependent manner in L6 myotubes. This activation, along with the increased phosphorylation of MEK3/6 (an upstream regulator of p38-MAPK), plays a critical role in AMPK-mediated glucose uptake [26].

### CUR and insulin resistance modulation

Insulin resistance (IR) plays a central role in PCOS pathogenesis, especially through the PI3 K and Akt signaling pathways. In a DHEA-induced PCOS animal model, CUR (100 and 200 mg/kg) significantly reduced fasting insulin levels and HOMA-IR, highlighting its ability to improve IR (P < 0.05). CUR's effect on IR is linked to its regulation of GLUT- 4 and estrogen receptor α (Erα) expression [27]. Additionally, it modulates IR via the PI3 K pathway [28]. CUR enhances GLUT4 expression, improving insulin sensitivity by facilitating glucose uptake [24]. In an estradiol-valerate-induced PCOS rat model (n = 36), CUR treatment (100 and 300 mg/kg; 14 days) significantly reduced insulin levels compared to the control (P < 0.001 and P < 0.05, respectively) [29]. By activating the PI3 K/Akt pathway, CUR promotes GLUT4 translocation to cell membranes, improving glucose uptake. It also reduces plasma levels of pro-inflammatory cytokines like TNF-α and IL- 6, contributing to improved insulin sensitivity and reduced obesity-induced insulin resistance [25].

### CUR and gut microbiota

Dysbiosis of the gut microbiome is often associated with PCOS, with testosterone playing a role in altering gut flora. It affects gut microbial enzymes directly and influences androgen receptors and immune regulation indirectly. Hyperandrogenism, a hallmark of PCOS, may induce gut microbiota imbalances. Animal studies have shown that letrozole, a common PCOS-inducing agent, alters gut microbiota by changing the abundance of *Bacteroidetes* and *Firmicutes*, and increasing the relative abundance of genera like *Coprococcus*, *Allobaculum*, *Bifidobacterium*, and *Ruminococcaceae*. Furthermore, altered gut microbiota can impact the lipopolysaccharide pathway, contributing to insulin resistance and obesity, common in PCOS [30]. CUR supplementation, when added to a high-fat diet (HFD), significantly altered the gut microbiota composition. In obese mice, CUR supplementation resulted in distinct differences in microbiome diversity, with a notable decrease in the relative abundance of *Ruminococcaceae*, *Burkholderiaceae, and Verrucomicrobia* phyla. On the other hand, CUR led to an increase in *Lactococcus*, *Turicibacter, and Parasutterella* genera, indicating potential beneficial shifts in the gut microbiota [31].

### CUR for PCOS treatment: focus on animal studies

Reddy et al. examined the effects of CUR on oxidative stress and lipid peroxidation in letrozole-induced female Wistar rats over 36 days. CUR (100 and 200 mg/kg) significantly increased catalase and superoxide dismutase (SOD) activity, and the higher dose (200 mg/kg) reduced TBARS levels. Both doses of CUR induced ovulation and led to cyst disappearance [19]. In estradiol valerate-induced PCOS rats (18 rats per group), CUR (100 and 300 mg/kg) treatment for 14 days significantly reduced necrotic hepatocyte count, interleukin- 6, TNF-α, and C-reactive protein levels (p < 0.05), as analyzed by one-way ANOVA. CUR treatment also increased estrogen levels, which had been depleted by aromatase inhibition [23]. In a study assessing CUR's impact on insulin resistance genes and GLUT- 4 in DHEA-induced PCOS rats, CUR (200 mg/kg) effectively restored normal body weight, insulin resistance, blood glucose, and serum insulin levels compared to the 100 mg/kg CUR and control groups (p < 0.05) [32].

### CUR for PCOS treatment: focus on clinical studies

A clinical trial involving 36 women with PCOS and 36 healthy women showed that CUR supplementation (1500 mg, three times daily for 3 months) significantly increased serum Glutathione Peroxidase (GPx) and SOD activity in women with PCOS (p ~ 0.045) [33]. In a triple blind study by Ghanbarzadeh-Ghashti and co-workers, 27 women with PCOS received CUR (500 mg twice daily for 3 months), which led to reduced serum testosterone (p = 0.13) and sex hormone binding globulin (SHBG) levels (p ~ 0.7), helping normalize menstrual irregularities, as analysed by ANOVA, Mann Whitney U-test, and independent T-test. [34]. A single-blind randomized trial with 15 women with PCOS treated with CUR (93 mg/kg, twice daily for 2 weeks) showed significantly decreased LH and FSH levels compared to the placebo group (*P* < 0.05). CUR also reduced fasting insulin, HOMA-IR, serum LDL, TC, and TG levels (*P* < 0.05), with statistical analysis by Shapiro–Wilk, Wilcoxon, Kruskal–Wallis, and Fisher’s exact tests [35]. Jamilian et al. (2020) reported that CUR (500 mg/day for 12 weeks) significantly reduced body weight and waist circumference in 30 women with PCOS compared to 30 healthy controls (*P* < 0.05). CUR also decreased serum insulin, total cholesterol (TC), LDL (P < 0.001), and glucose levels (*P* < 0.01), while increasing HDL (*P* < 0.001) and insulin sensitivity, analyzed using Kolmogorov–Smirnov and independent T-test [36].

### Formulation approaches explored for the CUR to treat PCOS

**Fig. 3:**
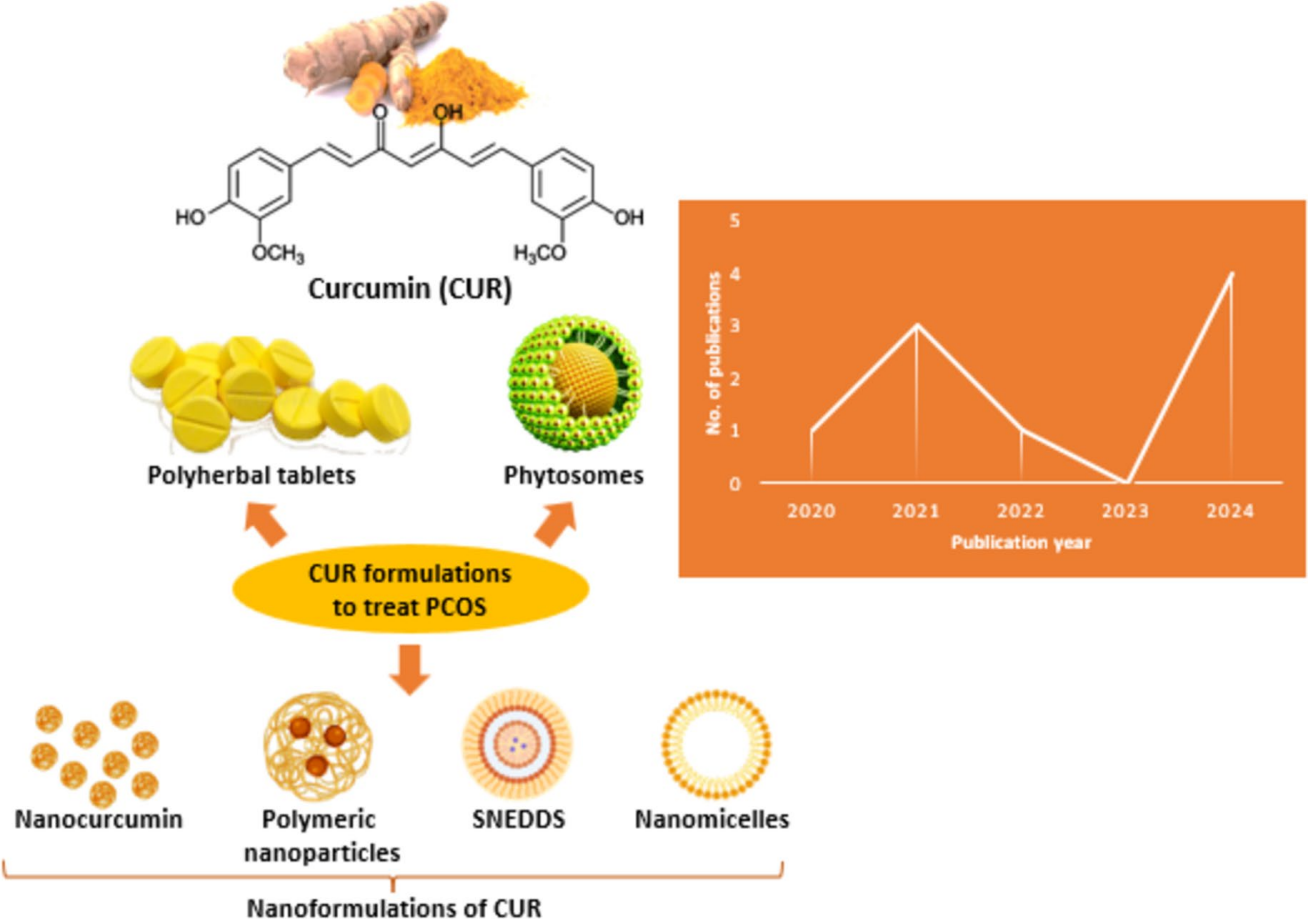
**a** Structure of CUR, **b** Formulations of CUR for PCOS treatment, and **c** Statistical representation showcasing the evolution of publications on formulations of CUR over time

The existing literature highlights various formulation approaches for enhancing the efficacy of CUR, including phytosomes [37], polyherbal tablets [38], nanoparticles [39, 40], and self-nanoemulsifying drug delivery system (SNEDDS) [41]. Figure 3 illustrates the formulation and publication trends on the use of CUR formulations to treat PCOS.

#### Polyherbal formulations containing CUR

**Fig. 4:**
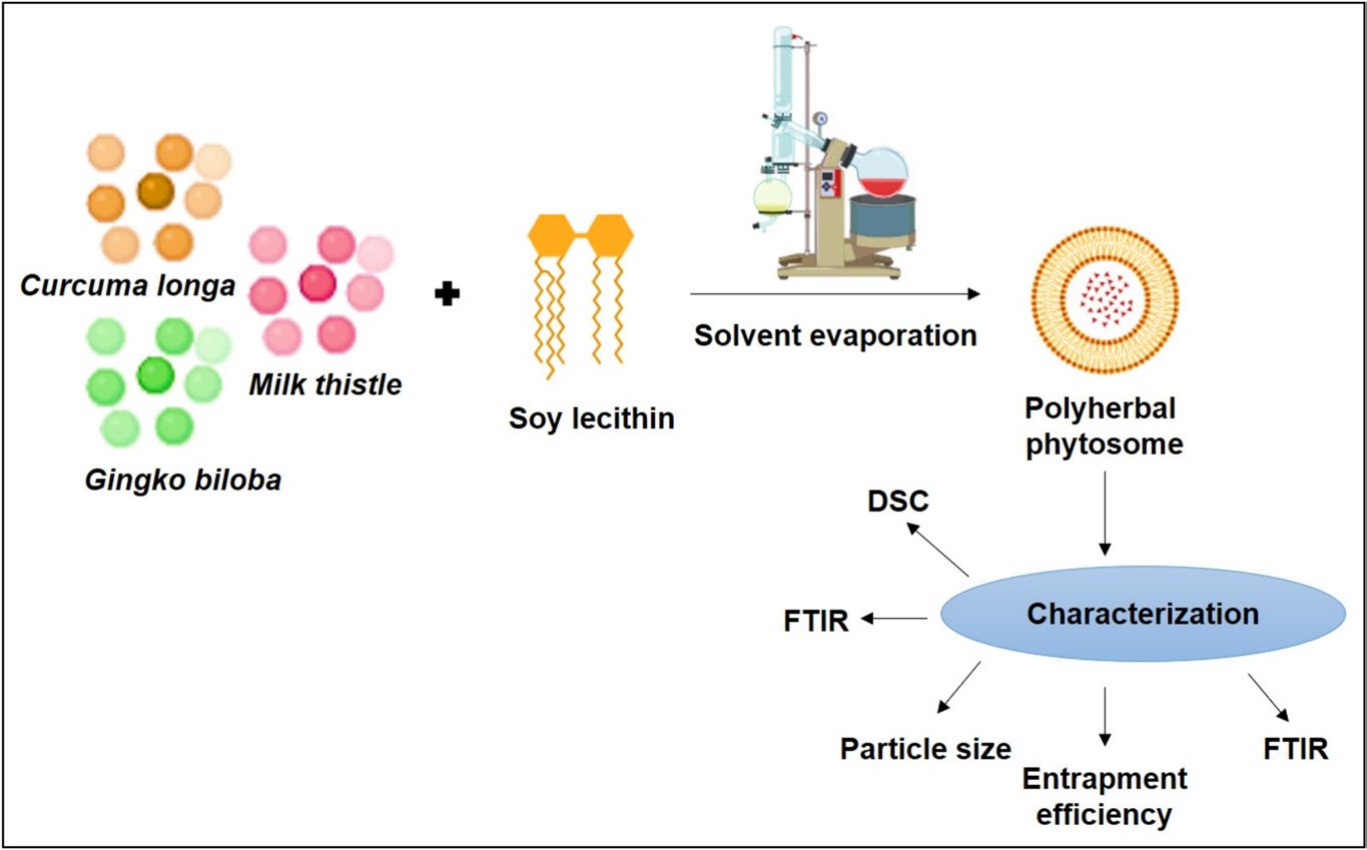
Schematic diagram of preparation and characterization of polyherbal phytosomes including CUR by solvent evaporation

Phytosomes of CUR, milk thistle, and *Ginkgo biloba* extracts using soy lecithin were prepared by Prema et al. (2024) (Fig. 4). The qualitative screening identified the presence of tannins, flavonoids, carbohydrates, and terpenoids. Various molar ratios of extract to lipid, including 1:1, 1:2, 2:1, 2:2, and 3:1 (w/w), were employed to prepare the phytosomes via the rotary evaporation method. Phytosomes with lower phospholipid content exhibited smaller particle sizes, with zeta potentials ranging from − 20.4 to − 29.6 mV. Among the formulations, the 2:2 ratio showed the highest entrapment efficiency of 65.9%, while the 2:1 ratio demonstrated 98% permeability. However, despite promising physicochemical properties, the study leaves an important gap by not exploring the potential therapeutic efficacy of these phytosomes in PCOS treatment through animal models or pathology assessments, highlighting an exciting area for future research [38].

**Fig. 5:**
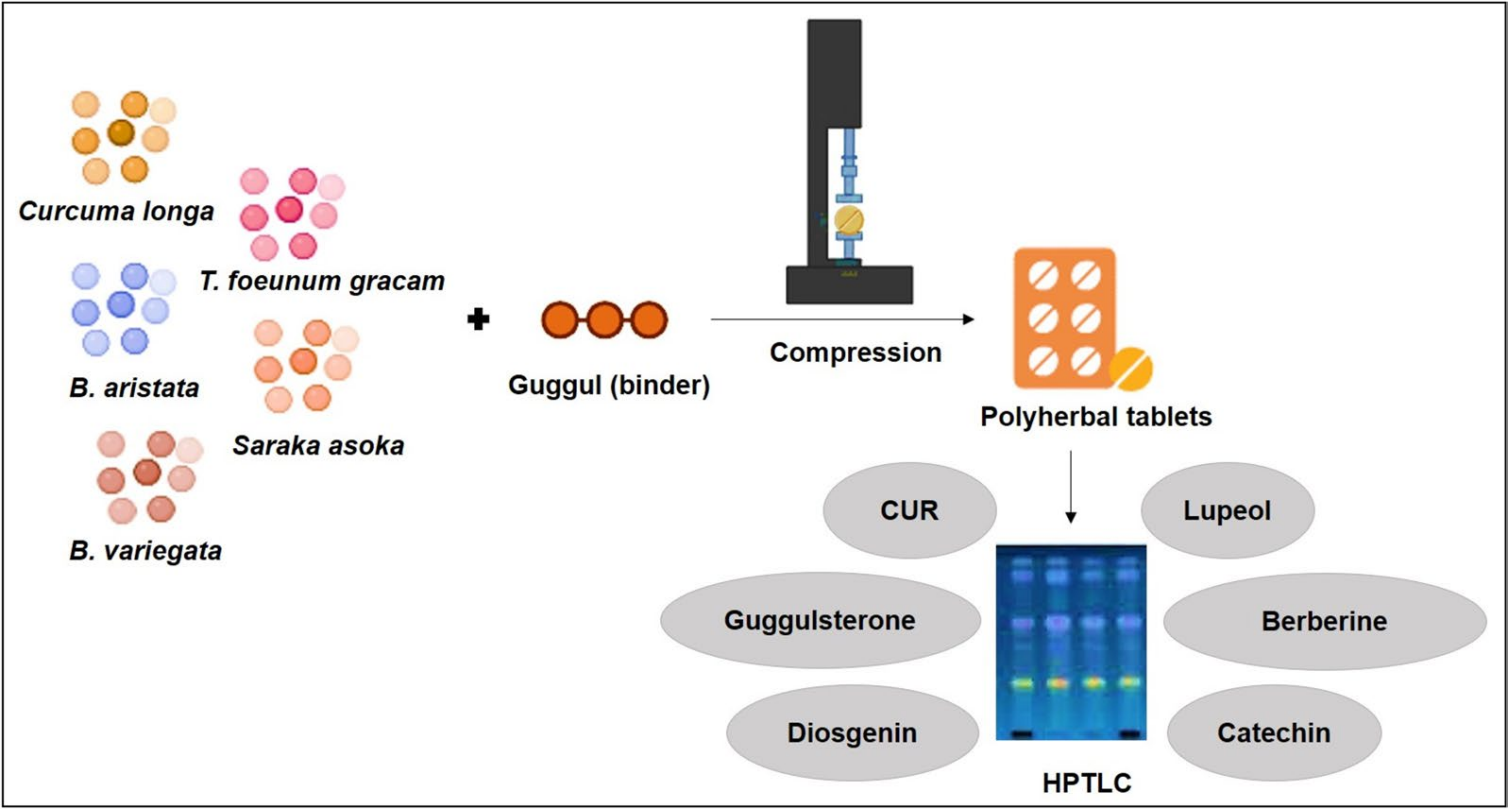
Schematic diagram of preparation and evaluation of polyherbal tablets by compression method

Prajapati and co-workers developed and assessed a polyherbal tablet containing extracts of *Berberis aristata*, *Bauhinia variegata*, *Trigonella foenum-graecum*, *Saraka asoka*, and *Curcuma longa* (Fig. 5). The tablets were prepared through compression using guggul as a binder. The quantitative screening, phytochemical, and physicochemical evaluations were conducted using HPTLC. Phytochemical analysis identified the presence of alkaloids, tannins, proteins, flavonoids, glycosides, carbohydrates, and terpenoids. HPTLC results confirmed the presence of key markers, including curcuminoids, berberine, catechin, lupeol, diosgenin, and guggulsterone. The tablets demonstrated stability at 40 °C and 75 ± 5% relative humidity for 3 months. Drug release after 2 h was above 90% [38]. However, a major limitation of this study is the lack of in vivo studies using a PCOS animal model or clinical trials to translate the preclinical findings into practical applications. Additionally, toxicity studies are essential to further assess the safety and efficacy of these polyherbal tablets before they can be considered for widespread use in PCOS treatment.

### Nano formulations

#### Nanoparticles

**Fig. 6:**
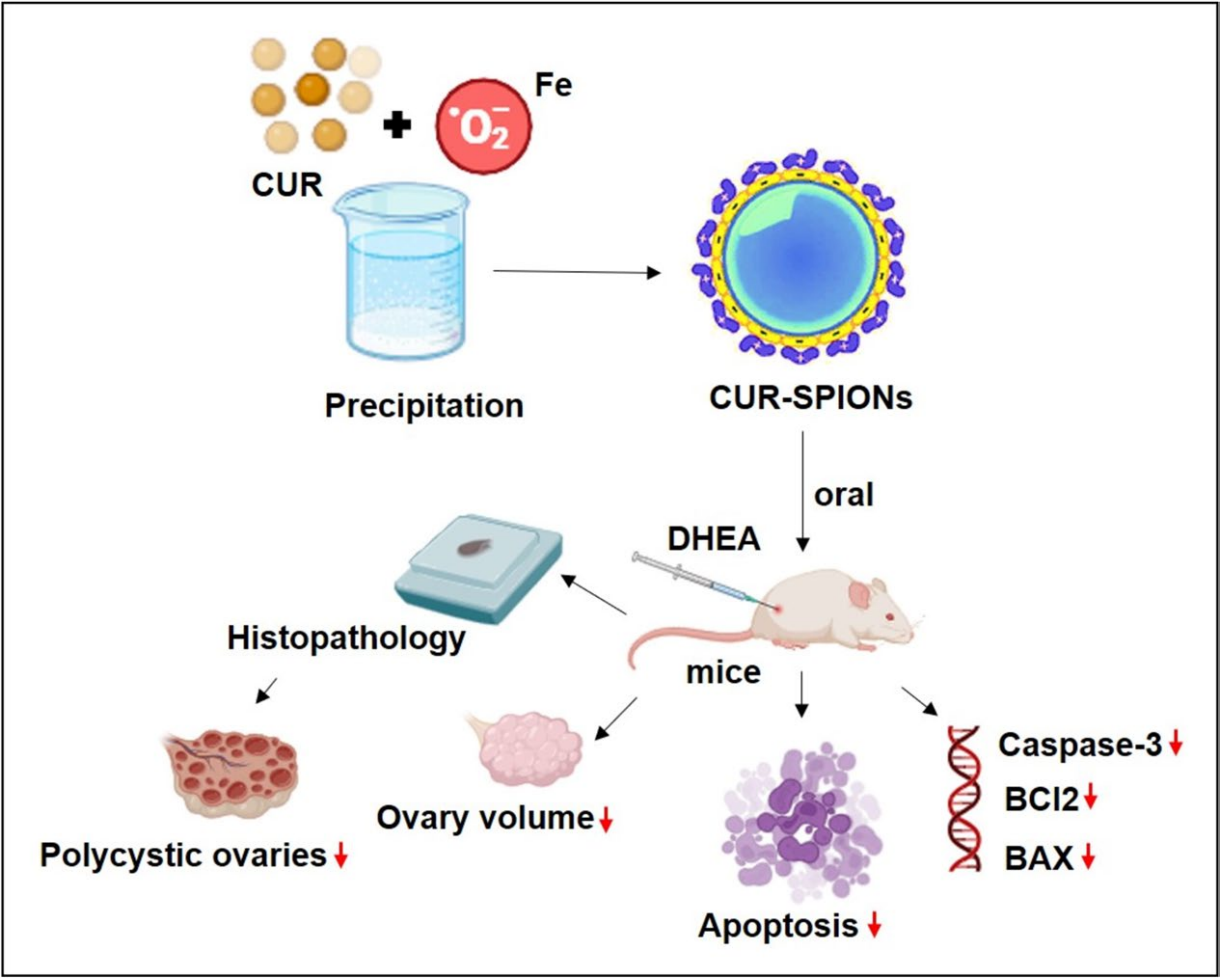
Schematic representation of preparation of CUR loaded superparamagnetic iron oxide nanoparticles (SPIONs) and its efficacy studies in DHEA induced PCOS mice

CUR-loaded superparamagnetic iron oxide nanoparticles (SPIONs) were synthesized using the precipitation method (Fig. 6). The formulation was tested in forty BALB-c prepubertal mice administered DHEA (6 mg/kg), with results compared to groups treated with CUR alone and controls using ANOVA and Tukey's post hoc test. Over 21 days, CUR treatment significantly reduced apoptosis in granulosa cells of PCOS mice (*P* < 0.05). CUR also downregulated BAX gene expression and upregulated Bcl2 expression, both of which are involved in apoptosis regulation in PCOS (*P* < 0.05). Caspase- 3 expression was markedly lower in granulosa cells of CUR-treated mice compared to the increased expression in the PCOS group (P < 0.05). Histopathological analysis revealed an increased number of primordial, primary, secondary, and antral follicles, as well as increased ovarian volume in DHEA-induced PCOS mice. However, CUR-SPIONs led to a significant reduction in follicle count and ovarian volume [39].

**Fig. 7:**
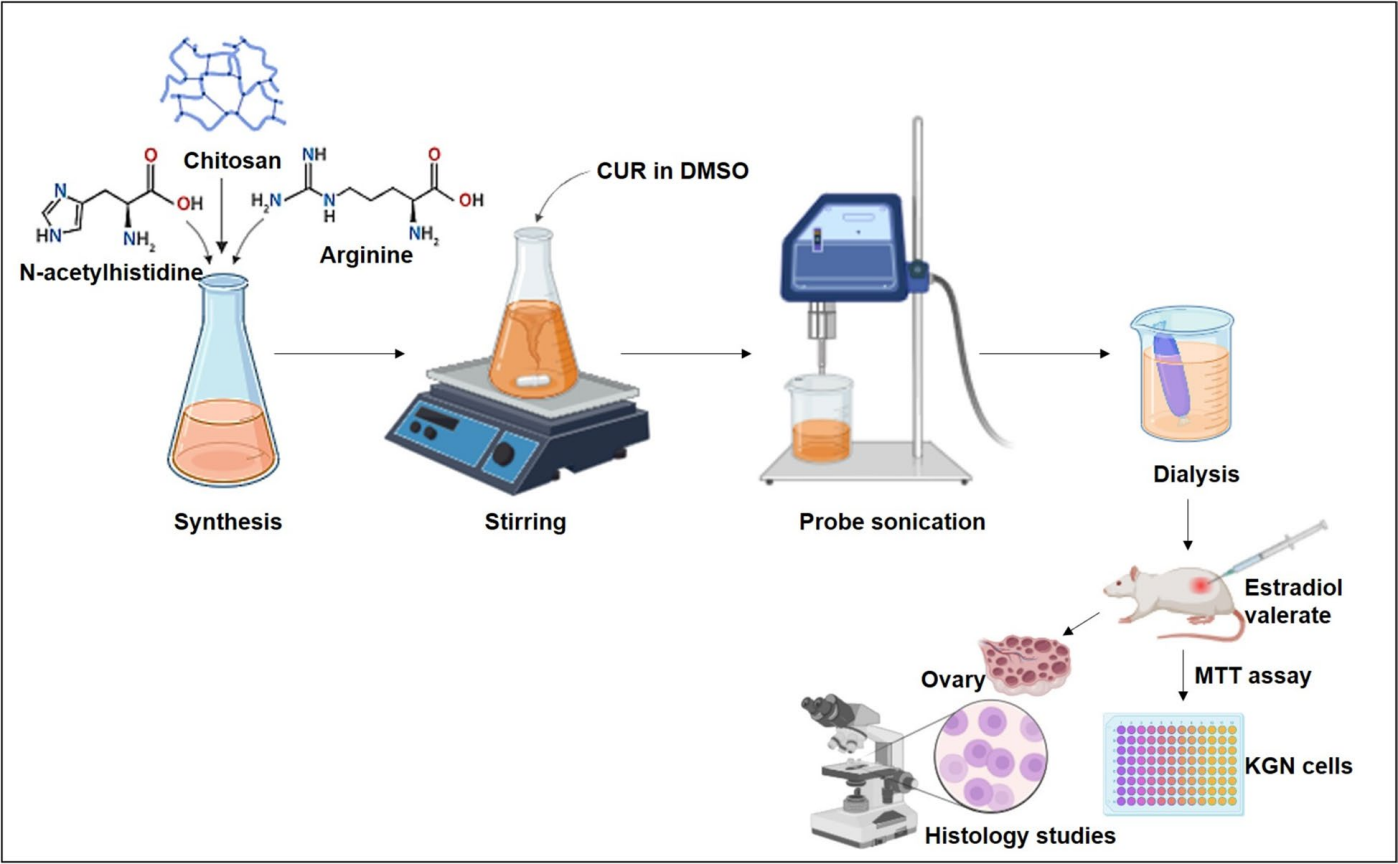
Schematic diagram of synthesis of chitosan conjugated self-assembled CUR nanoparticles by sonication and dialysis technique with its effects on animal and in vitro model of PCOS

Raja et al. developed chitosan-conjugated self-assembled CUR nanoparticles (50 mg/kg) using the sonication method (Fig. 7). These nanoparticles were characterized for their physicochemical properties and assessed for efficacy in estradiol valerate (2 mg/kg)-induced PCOS rats. The particle size and zeta potential were 200 nm and approximately 40 mV, indicating the stability of the nanoparticles. CUR release from the nanoparticles occurred in a sustained manner. Cytotoxicity tests in KGN cells (human granulosa cell line) using the MTT assay revealed IC50 values of 27.5 μmol/L for CUR nanoparticles and 17.3 μmol/L for free CUR. Further efficacy evaluation, with statistical analysis using one-way ANOVA and Bonferroni post hoc test, showed that CUR nanoparticles normalized the estrous cycle, increased serum FSH and progesterone levels, and reduced LH, prolactin, testosterone, and insulin (*P* < 0.05). Additionally, CUR nanoparticles improved ovarian morphology, promoting follicular development, increasing corpus luteum count, and reducing cystic follicles in the ovaries of rats [40].

#### Self-nanoemulsifying drug delivery system (SNEDDS)

SNEDDS are composed of lipids, surfactants, and co-surfactants that enhance solubility, membrane permeability, lymphatic absorption, and lipoprotein integration, with minimal side effects. In a study by Wahyuni et al., CUR-SNEDDS (25–100 mg/kg) demonstrated effectiveness in treating PCOS in rats induced by letrozole and a high-fructose diet (n = 6 each), as compared to MTF (20 mg/kg). Data were analyzed using Kruskal–Wallis and ANOVA tests. CUR-SNEDDS (50 and 100 mg/kg) significantly reduced fasting blood glucose, serum insulin, and HOMA-IR index (P < 0.05), while increasing GLUT- 4 expression, suggesting its potential as an insulin sensitizer [41].

#### Nanocurcumin (NanoCUR)

**Fig. 8:**
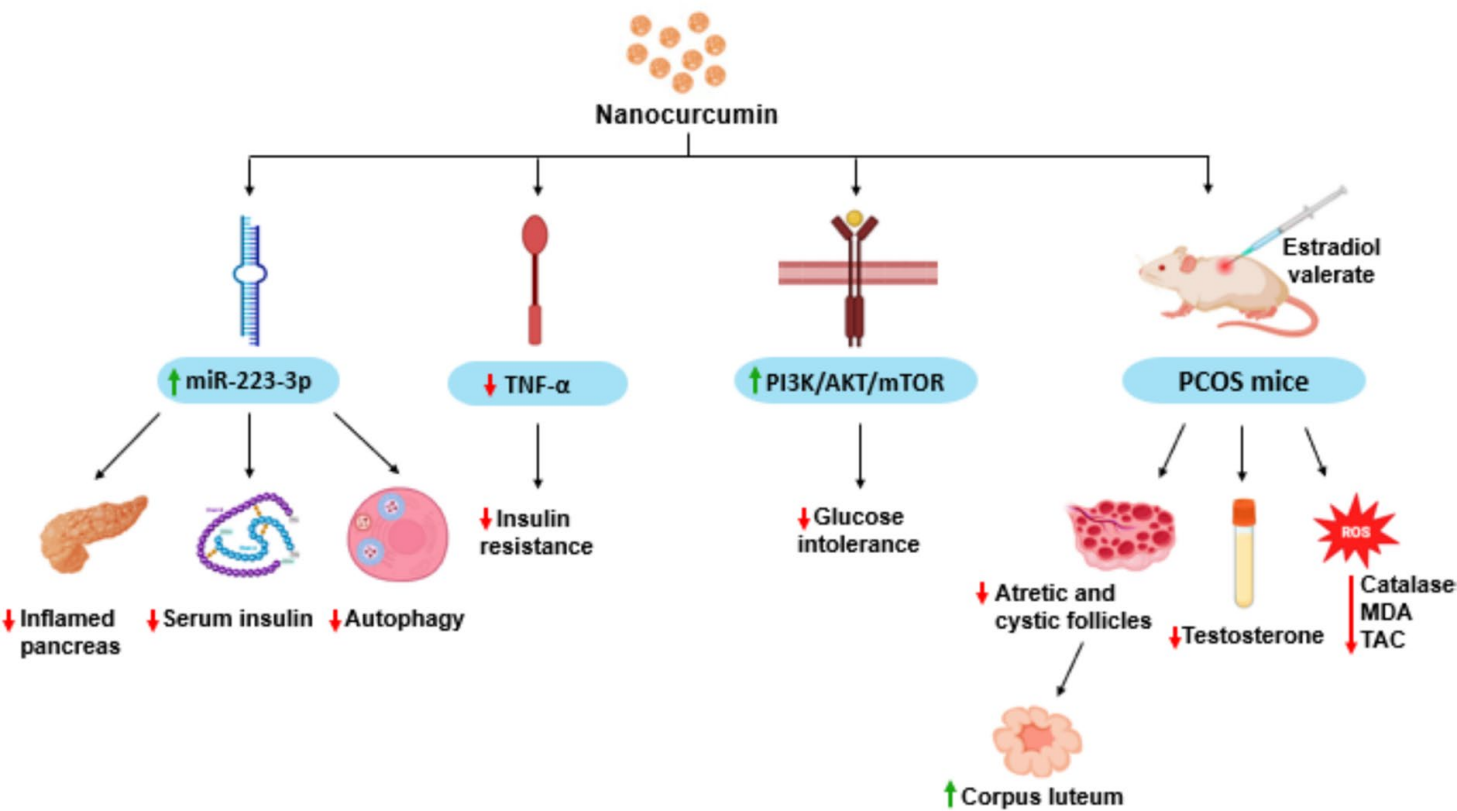
Nanocurcumin for the treatment of PCOS

NanoCUR’s potential for treating PCOS lies in its ability to enhance oral bioavailability by improving aqueous solubility and absorption [42]. Preclinical studies show that nanoCUR (25–200 mg/kg) reduces blood glucose, oxidative stress markers, inflammatory markers (including TNF-α), sex hormones, and insulin levels. Abuelezz et al. found that a 30% increase in miR- 223 - 3p expression regulates insulin, autophagy, and inflammatory pathways in the pancreas. In Letrozole-induced PCOS rats, nanoCUR (200 mg/kg) significantly increased NF-κB, LC3II, and p62 markers, confirming its molecular target. It also reduced IL- 6 (*P* < 0.05), MDA, GSH, and Catalase levels (*P* < 0.01) compared to control and Clomiphene-treated rats, as analyzed by one-way ANOVA with Tukey’s post hoc test. Additionally, nanoCUR alleviated insulin resistance and glucose intolerance by activating the PI3 K/AKT/mTOR pathway [43, 44]. Gharibeh et al. reported improved fertility in female mice after nanoCUR treatment. In estradiol valerate-induced PCOS mice, nanoCUR (12, 25, and 50 mg/kg) for 21 days reduced serum testosterone, MDA, catalase, and total antioxidant capacity. NanoCUR (25 and 50 mg/kg) also decreased the number of atretic and cystic follicles, increased corpus lutea count, and normalized ovarian morphology (Fig. 8) [45]. Despite significant progress in nanoformulations, challenges related to drug loading efficiency and toxicity still need to be addressed. The materials used in the synthesis and preparation of nanoparticles are expensive in comparison to herbal extracts and conventional dosage forms such as tablets and capsules. The European Union’s Novel Food Regulation requires a risk assessment for nanomaterials before they can be used as primary ingredients in products, ensuring their safety for human use [46]. In order to overcome these limitations, combination therapy of CUR with a herbal extract of a phytochemical is necessary to reduce the components of nanoparticulate system and reduce the toxicity of ingredients.

### Combination of CUR with other therapeutic compounds to treat PCOS

#### CUR and fecal microbiota transplant (FMT)

**Fig. 9:**
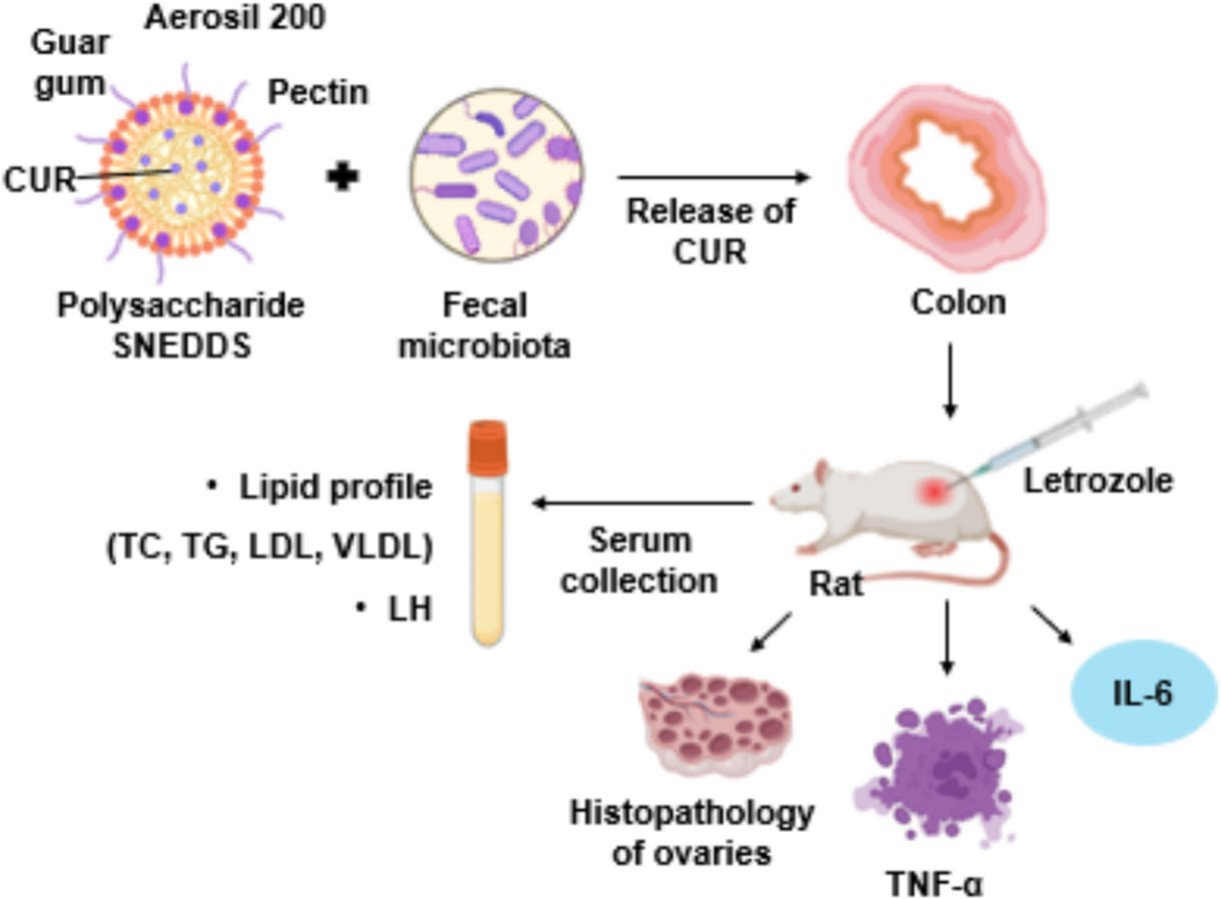
Schematic diagram for the preparation of polysaccharide-fecal microbiota based SNEDDS for colon targeted delivery of CUR and evaluation of its efficacy in letrozole induced PCOS rats

Dysbiosis of gut microbiota or DOGMA theory correlates gut microbiota and PCOS with obesity and high fat diet as key underlying factors [47]. There exists a well-established connection between PCOS and gut dysbiosis due to the role of gut microbiota in the regulation of inflammation, sex hormones, obesity, and insulin resistance. Any imbalance in sex hormone level leads to changes in the gut microbiome diversity, attributed to the estrogen gut microbiome axis. FMT improves serum estrogen, estrous cycle, and ovarian dysfunction [30]. In a study by Guo et al., the impact of gut microbiome composition on the PCOS pathogenesis was evaluated in letrozole-induced Sprague Dawley rats. Dunnett’s post-hoc test was applied to compare and analyse the difference in the microbiome composition between letrozole induced PCOS rats (*n* = 8) and control rats (*n* = 8). PCOS rats showed a decrease in *ruminococcus*, c*lostridium*, and *lactobacillus*, and an increase in *Prevotella* species (*P* < 0.05). FMT restored gut microbiota composition and estrous cycle in PCOS rats within 14 days [48]. CUR improves the gut microbial levels of *lactobacillus*, *bifidobacterium*, and butyrate producing bacteria while decreased *prevotellaceae*, *enterobacteria*, and *coriobacterales*. This highlights the anti-hyperlipidemic activity of CUR [49]. In a retracted article, Singh and co-workers prepared polysaccharide-fecal microbiota based SNEDDS for colon targeted delivery of CUR (25 mg/kg) (Fig. 9). They reported loading of CUR in SNEDDS to be 95.9%. The formulation markedly enhanced the dissolution rate by 2.3-fold and oral bioavailability by almost 13-folds than free CUR. Letrozole induced Wistar rats were selected as PCOS model to evaluate the efficacy of formulations and the data was analysed by one-way ANOVA. CUR-SNEDDS reduced body weight gain in letrozole induced PCOS rats. It decreased elevated testosterone, lipids such as TC, TG, LDL, and VLDL, LH, and inflammatory markers including TNF-α and IL- 6 levels (P < 0.05). It increased FSH and progesterone levels in serum (P < 0.05) indicative of improvement in the follicle development, ovarian morphology, and ovulation [50].

#### CUR and MTF

MTF is a USFDA-approved drug used to treat type II diabetes mellitus. In women with PCOS, it inhibits hepatic glucose production, enhances peripheral insulin sensitivity, and reduces glucose uptake in the intestine [51]. MTF has been shown to improve hyperandrogenism and menstrual irregularities, addressing core symptoms of PCOS. However, its gastrointestinal side effects and variable patient response highlight the need for alternative or adjunctive therapies. The combination of MTF and CUR presents a promising therapeutic approach, utilizing the complementary effects of both agents. MTF’s insulin-sensitizing action, combined with CUR’s anti-inflammatory and antioxidant properties, may offer a synergistic benefit in treating both the metabolic and reproductive abnormalities seen in PCOS. In a double-blind trial of 50 women with PCOS (according to Rotterdam Consensus criteria), the combination therapy led to greater reductions in LDL, TC, and TG levels, while increasing HDL. It also significantly improved glucose metabolism markers and reduced body weight and BMI. Additionally, testosterone levels decreased, and FSH and LH levels improved (P < 0.05) [52].

While MTF alone has a 6–40% chance of causing gastrointestinal side effects such as nausea, vomiting, and diarrhea (50), a study by Shah and Shrivastava in letrozole-induced PCOS mice compared the effects of CUR extract (175 mg/kg) and MTF (150 mg/kg). The results showed that CUR extract more effectively alleviated metabolic-endocrine dysfunction, inflammation, and oxidative stress compared to MTF [53]. The combination of CUR and MTF was further evaluated in a clinical trial by Sohrevardi et al. (2021), which included 100 women with PCOS. The study found that CUR delivered through nano micelles (80 mg/day) was more effective than 500 mg of MTF (taken three times daily) in reducing serum testosterone, insulin, HOMA-IR, TC, TG, and LDL levels (P < 0.05). After 12 weeks of treatment, the CUR group had significantly lower LDL, HDL, TC, and TG levels compared to the MTF group (*P* < 0.05) [54].

Kekatpure et al. conducted a double-blind, randomized, placebo-controlled trial to evaluate the combination of MTF (1 g) and CUR (1.2 g) for cancer prevention. Their hypothesis suggested that this combination could downregulate the NF-kB pathway, which is also implicated in PCOS. No serious adverse effects were noted after 9 weeks of treatment [55]. In a separate study with Wistar rats, the combination improved antioxidant activity and dyslipidemia, significantly reducing plasma glucose, triglycerides, cholesterol, and TBARS, with results analyzed using ANOVA and the Student–Newman–Keuls te(st [54]. Although no major side effects were reported in PCOS treatments, a rare case of cachexia was observed in diabetic mice treated with the combination via intraperitoneal injections [56]. Further research demonstrated that the Met-Cur combination, when delivered via chitosan-alginate nanoparticles, produced the strongest anti-inflammatory effects. The nanoparticles were well-tolerated at therapeutic doses without significant side effects [57].

#### CUR and Sirtuin- 1

Silent Information Regulator- 1 or Sirtuin- 1 (SIRT- 1) modulates mitochondrial function, glucose, and lipid metabolism through peroxisome proliferator-activated receptor-gamma (PPAR-γ), mitochondrial transcription Factor A (TFAM), and nuclear respiratory factor activation, which are linked to insulin resistance [58]. In a clinical trial with 36 women with PCOS, CUR treatment showed a non-significant increase in SIRT1 expression. SIRT1 regulates oxidative stress and prevents DNA damage via p53, while promoting lipid oxidation through PPAR-α activation and deacetylation of PGC- 1α. It also affects insulin and glucose balance via PGC- 1α. CUR inhibits hepatic gluconeogenesis via the AMPK pathway, lowering blood glucose. Additionally, CUR increased serum GPx and SOD activity (p = 0.01) and upregulated PGC- 1α expression, indicating a positive correlation between SIRT1 and PGC- 1α. The study concluded that CUR's antioxidant activity is driven by SIRT1-mediated deacetylation of PGC- 1α [33]. Although the study reported effectiveness of combination therapy, any potential adverse effects were not reported.

#### CUR and zinc

Zinc supplementation (50 mg/day) for 8 weeks in women with PCOS has been shown to significantly increase serum zinc levels compared to healthy controls, while also lowering blood glucose and insulin levels. This effect is thought to result from zinc's antioxidant properties, its role in stabilizing insulin hexamers, and enhancing insulin binding to hepatocyte membranes [59]. However, in a retracted article by Jamilian et al. it was observed that although zinc improved hirsutism, alopecia, and malondialdehyde (MDA) levels, it had no significant impact on hormonal profiles or markers of inflammation and oxidative stress [60]. Abd-Alqader et al. examined the combined effects of CUR (CUR) and zinc on hormonal profiles in letrozole-induced PCOS rats. Their study revealed that CUR (200 mg/kg), with or without zinc (30 mg/kg), significantly increased progesterone and estrogen levels while reducing elevated testosterone, prolactin, LH, and FSH levels. The combination of CUR and zinc showed a greater potential to normalize hormonal imbalances than CUR, zinc, or MTF alone [18]. A randomized clinical trial by Karandish et al. evaluated the combined effects of CUR (500 mg/day) and zinc supplementation (30 mg/day) over three months in pre-diabetic and overweight individuals. The study found no serious adverse effects and reported improvements in serum lipid levels, glucose control, antioxidant activity, and inflammation markers [61]. These findings suggest that the CUR and zinc combination may offer significant benefits for managing PCOS, but further clinical studies are necessary to confirm their synergistic effects and potential as a reliable treatment option for the condition.

#### CUR and Ziziphora extract

Ziziphora plant extract contains Pulegone, an active compound with potential anti-inflammatory properties. It inhibits lipid peroxidation, reduces oxidative stress, and subsequently lowers inflammation [62]. Nabiuni et al. studied the impact of Ziziphora extract on inflammatory markers like TNF-α, IL- 6, and CRP, as well as ovarian morphology. In Ziziphora-treated rats, ovarian sections showed a significant increase in corpus luteum and a decrease in granulosa layer thickness. The extract also reduced IL- 6 and CRP levels, while TNF-α expression was higher in the follicular fluid, granulosa cells, and ovarian cysts compared to the control group [63]. Similarly, a 14-day treatment with CUR (600 mg/kg, IP) and Ziziphora (300 mg/kg) resulted in significant reductions in body weight, serum testosterone, estradiol, and LH, with increased progesterone and FSH levels in PCOS rats, as analyzed by one-way ANOVA. This combination also lowered TC, TG, LDL, and VLDL levels, reduced cystic follicles, and improved ovarian morphology, with no adverse effects observed upon co-delivery [64]. While the combination of CUR and Ziziphora has shown promise in improving serum lipid and hormonal levels in PCOS, further well-designed clinical trials are needed to confirm their synergistic effectiveness. Larger studies with diverse patient groups are essential to validate their safety, efficacy, and long-term benefits, ensuring this combination becomes a reliable treatment option for PCOS.

#### CUR and Emblica officinalis

In Ayurveda, CUR and *Emblica officinalis* are considered first-line treatments for diabetes. Gupte et al., in their exploratory clinical trial, hypothesized that these herbs could also improve insulin sensitivity in women with PCOS. Their study found that a traditional formulation of CUR and *Emblica officinalis* significantly reduced total cholesterol (TC), insulin, HOMA-IR, and blood glucose levels compared to the control and MTF, effectively alleviating metabolic abnormalities. Additionally, the combination decreased serum testosterone, LH, and IL- 6 levels while increasing TNF-α. It also helped normalize the estrous cycle and reduced ovarian volume in women with PCOS [65]. *Emblica officinalis*’s potential in PCOS treatment was further supported by molecular docking studies performed by Kamboj et al., which investigated its ability to inhibit CYP17-α, an enzyme critical for steroid hormone synthesis, including estradiol, DHEA, cortisol, and testosterone. The study showed that *Emblica officinalis*, along with *Terminalia bellirica* and *Terminalia chebula*, had significant inhibitory activity against CYP17-α, with binding affinities ranging from − 3.7 to − 9.5. Specifically, key constituents of *Emblica officinalis* such as phyllemblin, rutin, chebulagic acid, and vitamin C exhibited strong binding affinities (− 5.5, − 9.5, − 8.2, and − 52, respectively), suggesting their efficacy in treating PCOS [66]. These findings highlight the potential of CUR and *Emblica officinalis* as an effective herbal therapy for managing PCOS, particularly in addressing metabolic and hormonal imbalances. However, although one study has reported promising results using this combination, further clinical trials and in vivo studies involving animal models of PCOS are needed to validate its efficacy for the treatment of PCOS and establish its safety profile.

#### CUR and teupolioside

Teupolioside is a phenylpropanoid glycoside derived from Ajuga reptans used in the treatment of cardiovascular diseases, respiratory tract disorders, and skin ailments. Teupolioside contains caffeic acid with an ability to suppress DHT and testosterone activity by primarily acting 5α-reductase, responsible in treating adolescent acne and androgenetic alopecia. The combination of CUR and teupolioside treats hyperandrogenism associated with PCOS [67]. However, further clinical trials are necessary to confirm its efficacy and safety for PCOS treatment.

#### CUR and vitamin D

Vitamin D deficiency is common in women with PCOS, affecting 20%–48% of the population. Vitamin D plays a crucial role in glucose metabolism, insulin synthesis, and secretion, as well as in regulating insulin receptor expression and reducing proinflammatory cytokines. Its effects on reproductive and metabolic abnormalities in PCOS are largely mediated by insulin resistance, which contributes to hyperandrogenism and decreased synthesis of sex hormone-binding globulin [68]. A meta-analysis by Zhang et al. found that Vitamin D supplementation in women with PCOS significantly increased Vitamin D levels, improved endometrial thickness, and reduced total cholesterol (TC), serum testosterone, and parathyroid hormone levels. Additionally, it lowered C-reactive protein (CRP), an inflammatory marker, suggesting that Vitamin D supplementation is beneficial for managing PCOS [69]. In support of these findings, Arabnezhad et al. conducted a randomized clinical study to assess the effect of CUR on Vitamin D levels in women with dysmenorrhea and premenopausal syndrome. Statistically tested by Mann–Whitney and Wilcoxon signed rank tests, CUR supplementation significantly increased serum Vitamin D levels compared to the control group (P < 0.05). However, CUR did not affect blood glucose or lipid profiles in women with PCOS, possibly due to underlying conditions like acute coronary disease and cognitive impairment. Due to the small sample size and short duration of the study, these findings should be interpreted with caution. While CUR improved Vitamin D levels, its effectiveness in addressing Vitamin D deficiency in PCOS requires further investigation [70].

### Recent patents on CUR for PCOS treatment

Two patents have been filed for CUR in treating PCOS. One patent combines sinomenine and CUR as active ingredients, with a sinomenine-to-CUR ratio ranging from 1:3.5 to 1:4.2. The formulation involves mixing sinomenine, CUR, hydroxypropyl cellulose, and mannitol, followed by granulation with a 10 mg/mL gelatin solution. The mixture is sieved, dried, and blended with lactose and talcum powder before being compressed into tablets. In DHEA-induced PCOS rats, this combination significantly improved serum insulin levels, alleviating insulin resistance [71]. Another patent discloses a formulation enriched with bisdemethoxycurcumin (20–80% w/w), demethoxycurcumin (10–35% w/w), and CUR (10–50% w/w). The tablet includes excipients such as microcrystalline cellulose, colloidal silicon dioxide, magnesium stearate, BioPerine, polyvinylpyrrolidone, starch, hydroxypropyl methyl cellulose, and hydroxypropyl cellulose. The capsule formulation contains active ingredients with microcrystalline cellulose and BioPerine. The powder form includes the active ingredients and BioPerine, while the gummy formulation contains the active ingredients, BioPerine, gelatin, refined sugar, glucose corn syrup, citric acid, lactic acid, water, mango flavor, and tartaric acid. The candy formulation includes the active ingredients, BioPerine, sucrose, liquid glucose, flavoring agents, menthol, acidulants, and purified water. In the letrozole-induced PCOS animal model, bisdemethoxycurcumin significantly reduced MDA levels, as well as elevated levels of advanced glycation end products and pro-inflammatory markers [72]. The patent details of these formulations are listed in Table 2.

**Table 2:** Patents on CUR for the treatment of PCOS

Sl no	Patent no	Title	Description	Reference
1	CN116687931B	Medicine for treating PCOS and application thereof	Concomitant administration of CUR and sinomenine normalized irregular estrous cycle in DHEA induced PCOS rats. The combination also improved insulin resistance	[71]
2	CA3213258 A1	Compositions for management of PCOS	The combination of bidsemothycurcumin (20–80%w/w), demethoxycurcumin (10–35%w/w), and CUR (30–50%w/w) decreased elevated body weight, ovary weight, thyroid stimulating hormone, serum testosterone, and glucose in letrozole induced PCOS mice. It also reduced oxidative stress, insulin resistance, hyperglycemia, hyperlipidemia, and gut dysbiosis	[72]

### CUR: Safety profile, toxicity concerns, and the impact of bioenhancers

CUR, known for its therapeutic benefits in various diseases, is generally considered safe when taken at doses of up to 3 mg/kg/day, as recommended by bodies such as the US Food and Drug Administration (FDA), the Joint United Nations World Health Organization Expert Committee on Food Additives (JECFA), and the European Food Safety Authority (EFSA) [73–75]. However, while CUR's benefits are well-documented in vitro, some studies suggest potential toxicity, particularly at higher concentrations (2.5–5 µg/mL), where DNA damage in both mitochondrial and nuclear genomes has been observed, raising concerns about its carcinogenic potential. A 1993 National Toxicology Program report also flagged the toxic and carcinogenic properties of turmeric oleoresin, a turmeric extract, highlighting the need for caution [76].

### Human Clinical Trials

In humans, CUR supplementation may cause liver toxicity in individuals with preexisting liver conditions or those on certain medications. Prolonged use can also lead to skin issues and stomach ulcers [77]. In humans, CUR supplementation may cause liver toxicity in individuals with preexisting liver conditions or those on certain medications. Prolonged use can also lead to skin issues and stomach ulcers (69). Clinical trials have linked doses of 0.45–3.6 g/day with nausea, diarrhoea, and increased serum enzyme levels, such as alkaline phosphatase and lactate dehydrogenase. For patients with high-risk lesions, doses exceeding 8 g/day are often unfeasible due to the large tablet volume [78]. Higher doses may lead to gastrointestinal discomfort, which can be alleviated by consuming CUR with meals. 0.45 to 12 g CUR may cause nausea, diarrhoea, headache, rush, yellow stool, and increased levels of both serum alkaline phosphatase and lactate dehydrogenase [79]. CUR doses up to 12 g/day are typically well tolerated in humans, though surpassing 8 g may prove challenging due to the large volume of the compound [80]. In prostate cancer patients, daily supplementation of 6,000 mg of CUR has been found safe and well-tolerated, with no adverse effects reported [81]. While some studies indicate CUR is undetectable in serum at doses under 4 g, others have found it in both serum and urine at lower amounts. Additionally, CUR has been detected in colorectal tissue at doses as low as 3.6 g, suggesting the gut may be a viable target for localized treatment [80]. However, supplementation of 0.9–3.6 g/day for up to four months can lead to side effects, including nausea, diarrhoea, and increased levels of alkaline phosphatase and lactate dehydrogenase [82]. High doses (> 500 mg/kg/day) may increase risks of kidney calculus, skin irritation, chronic anemia, and even drug-induced liver damage [83].

### In vivo and animal studies

Animal studies have largely supported CUR’s safety. For instance, a high-dose study (3500 mg/kg) in monkeys and dogs found no significant adverse effects, and no cytotoxicity was noted in liver or other tissues at doses up to 1000 mg/kg/day in rats [83]. Bhatt et al. conducted a study in male Sprague Dawley rats, where chemically modified CUR (CUR 2.24) was administered via oral gavage at doses of 50 mg/kg, 100 mg/kg, 500 mg/kg, and 1000 mg/kg per day for five days. No cytotoxic effects were observed in the liver or other tissues of lungs, spleen, heart, colon, and kidneys [84]. In studies involving formulated nanoparticles, a single dose equivalent to 2000 mg/kg of CUR was found to be safe when administered orally to Swiss albino mice. Sub-acute toxicity studies showed no animal fatalities across all groups during the study period. Genotoxicity assessments revealed no evidence of chromosomal or DNA damage [85]. Similarly, CUR was well tolerated in Swiss albino mice at doses equivalent to 2000 mg/kg [85], further suggesting that CUR’s safety is dose-dependent.

### Role of bioenhancers

Herbal bioenhancers are widely used in modern medicine due to their safety, rapid absorption, and minimal side effects. They enhance drug bioavailability, improving efficacy, plasma levels, and reducing required doses, treatment time, and toxicity [86]. However, excessive bioenhancement can lead to CUR accumulation and toxicity, as seen with CUR and 40 mg/kg piperine co-administration, which raises tissue concentration and diminishes CUR’s benefits [87].

Piperine, a well-known bioenhancer, has demonstrated therapeutic benefits in conditions like diabetes, arthritis, cancer, and cardiovascular diseases. Other bioenhancers such as thymoquinone, quercetin, C. carvi, C. cyminum, glycyrrhizin, niaziridin, naringin, and genistein improve oral bioavailability by inhibiting the P-glycoprotein efflux pump and metabolic enzymes, which reduces drug dosages and toxicity [88]. In an investigational trial, Shukla et al. demonstrated that lysergol, a bioenhancer for CUR, increased permeability and improved pharmacokinetic parameters by 3.3-fold compared to CUR alone by inhibiting the P-gp efflux pump and Phase II metabolic enzymes [89]. Pinisetti et al. also showed that quercetin (50 mg) enhanced CUR's bioavailability by inhibiting the P-gp efflux pump and metabolic enzymes, leading to a 7.2-fold increase in CUR absorption in the intestine within an hour [90]. Chidambaram et al. developed CUR-loaded nanoparticles with bioenhancers such as quercetin, piperine, and silibinin that overcame CUR’s solubility issues and boosted anticancer effects, showing superior results compared to pure CUR (84). Bioenhancers effectively improve CUR's bioavailability, therapeutic efficacy, and safety [91]. Ultimately, bioenhancers can improve CUR’s permeability, oral bioavailability, and therapeutic efficacy while mitigating toxicity.

## Conclusion with a future view

In summary, CUR establishes significant potential as a therapeutic compound for treating PCOS due to its multifaceted pharmacological activities including antioxidant, anti-inflammatory, anti-hyperlipidemic, and insulin-sensitizing effects. This review highlights the diverse mechanisms through which CUR alleviates PCOS symptoms, such as hormonal imbalance, lipid metabolism dysfunction, insulin resistance, hyperglycemia, oxidative stress, and inflammation. Existing evidence from preclinical and clinical investigation recommends that CUR can offer comparable effects to conventional therapy such as MTF, with additional advantages associated to fewer adverse effects and complementary therapeutic benefits. CUR formulations play a vital role in enhancing its therapeutic effectiveness, as its poor aqueous solubility and oral bioavailability incumbers clinical applications. Novel formulation approaches such as polyherbal combinations, nanotechnology-based drug delivery system, and self-nanoemulsifying delivery systems, offer potential benefit in improving CUR’s solubility, bioavailability, and therapeutic outcome. There is a need for an extensive clinical study in larger population to establish safety and efficacy of CUR formulations. Future research should consider long-term benefits, including effects on fertility, mental, reproductive, and metabolic health, and ultimately, quality of life in women suffering from PCOS. Investigation of combinatorial treatment synergizing CUR effect with various therapeutic or dietary compounds might deliver further benefits. The targeted delivery approach for CUR should focus on the molecular pathways of PCOS through which it acts on PCOS. Preclinical studies and a few clinical investigations have highlighted the impact of combining CUR with other active molecules. However, formulation approaches combining CUR with novel or existing compounds such as MTF, vitamin D, polyphenols, sirtuin- 1 activators, dipeptidyl peptidase- 4 inhibitors, and other polyphenols could address heterogenous features of PCOS more efficiently than individual drug therapy. It is essential to bridge the gap between promising animal study results and their application in humans by conducting clinical trials with women who have PCOS. These trials, conducted across various healthcare settings, are crucial to fully understand the safety, effectiveness, and tolerance of treatments. By gathering real-world data, we can better determine the appropriate dosage and ensure these therapies are both safe and effective for PCOS management. With the rising number of women affected by PCOS, it’s more important than ever to move from preclinical research to human trial. Moreover, regulatory approval of these formulations is of utmost importance before introducing CUR formulations into clinical setting for management of PCOS. In order for the scale-up and translation of research ideas into clinical practice, collaboration between clinicians, researchers, industrialists, and regulatory bodies is essential. CUR is potential in treating PCOS with multiple effects. Continual research into formulation development is crucial to further improve efficacy of CUR while offering novel avenues to treat this complex syndrome. In the future, personalized therapies tailored to individual patient profiles will likely play a crucial role in enhancing the impact of CUR formulations. Large-scale clinical trials, incorporating personalized approaches and diverse treatment regimens, will provide a more comprehensive understanding of CUR’s potential in managing PCOS. By integrating personalized medicine with large-scale studies, the precision and efficacy of treatments can be significantly improved, ultimately leading to better outcomes for women suffering from PCOS.

## Data Availability

No datasets were generated or analysed during the current study.
